# SVM-Based Normal Pressure Hydrocephalus Detection

**DOI:** 10.1007/s00062-020-00993-0

**Published:** 2021-01-26

**Authors:** Alexander Rau, Suam Kim, Shan Yang, Marco Reisert, Elias Kellner, Ikram Eda Duman, Bram Stieltjes, Marc Hohenhaus, Jürgen Beck, Horst Urbach, Karl Egger

**Affiliations:** 1 Department of Neuroradiology, Medical Center, University of Freiburg, Breisacher Str. 64, 79106 Freiburg, Germany; 2 Department of Neurosurgery, Medical Center, University of Freiburg, Freiburg, Germany; 3 Department of Research and Analysis, University Hospital Basel, University of Basel, Basel, Switzerland; 4 Medical Physics, Department of Radiology, Medical Center, University of Freiburg, Freiburg, Germany

**Keywords:** Support vector machine, Artificial intelligence, Machine learning, CSF shunt, Normal pressure hydrocephalus

## Abstract

**Background and Purpose:**

As magnetic resonance imaging (MRI) signs of normal pressure hydrocephalus (NPH) may precede clinical symptoms we sought to evaluate an algorithm that automatically detects this pattern.

**Methods:**

A support vector machine (SVM) was trained in 30 NPH patients treated with ventriculoperitoneal shunts and 30 healthy controls. For comparison, four neuroradiologists visually assessed sagittal MPRAGE images and graded them as no NPH pattern, possible NPH pattern, or definite NPH pattern.

**Results:**

Human accuracy to visually detect a NPH was between 0.85 and 0.97. Interobserver agreement was substantial (κ = 0.656). Accuracy of the SVM algorithm was 0.93 and AUROC 0.99. Among 272 prespecified regions, gray matter and CSF volumes of both caudate, the right parietal operculum, the left basal forebrain, and the 4th ventricle showed the highest discriminative power to separate a NPH and a no NPH pattern.

**Conclusion:**

A NPH pattern can be reliably detected using a support vector machine (SVM). Its role in the work-up of asymptomatic patients or neurodegenerative disease has to be evaluated.

## Introduction

Normal pressure hydrocephalus (NPH) is a brain disorder in which excess cerebrospinal fluid (CSF) accumulates in the subarachnoid and ventricular spaces. It is defined as increased CSF volume without continuous elevation of the intracranial pressure, whereas the complete pathomechanism is still unclear [1]. A resulting ventricular enlargement can disrupt and damage surrounding brain tissue, leading to gait disturbance, urinary incontinence and dementia, a constellation known as Hakim triad [2]. The NPH has an overall prevalence of 10.2–31.4/100,000, with an increase to 5900/100,000 in the population of patients older than 80 years [1] and is therefore of high health care relevance.

In some cases, NPH is caused by other brain disorders such as a tumor, head injury, hemorrhage, infection or inflammation. Yet in most cases, the cause of excess fluid remains unknown. Studies indicate that the development of NPH underlies a prolonged process since radiographic findings show volumetric changes 3 or more years before the onset of clinical symptoms [3].

Despite ongoing efforts, neither the pathophysiology of NPH nor the mechanics of the improvement after standard treatment with surgical CSF shunting are completely understood. The diagnosis of NPH is dependent on several clinical and radiological factors and is complicated by a variable degree of symptom presentation as well as neurodegenerative comorbidities [4]. Given the elusiveness of the disease, successful treatment with clinical improvement after shunting can be considered a reliable confirmation of the diagnosis, although 20–40% of CSF shunted patients do not significantly benefit [5].

Imaging used for the diagnosis of neurodegenerative diseases serves two functions: it should reveal treatable causes and contribute to the etiological differentiation of primary dementia disorders [6]. Treatable diseases such as tumor and subdural hematoma are underlying causes in less than 5% of patients with neurodegenerative symptoms, while nonocclusive hydrocephalus (including NPH) is more commonly found [7, 8].

In imaging, the most reliable sign for detecting NPH is a disproportional enlargement of the ventricles and the Sylvian fissure (disproportionately enlarged subarachnoid space hydrocephalus, DESH) in comparison to the constricted CSF spaces in the high convexities of the brain [9]. While ventriculomegaly is commonly found in studies as a sensitive but nonspecific NPH sign, the tight high convexity (THC) is highly specific [10]. Another parameter used in clinical routine is the callosal angle, which is decreased in patients with NPH [11]. In a recent study, the classification of THC using an automated machine learning approach showed an excellent performance with an AUROC of up to 0.99 (false negative ≈2%, false positive ≈5%) [12]. This high AUROC was achieved by selecting the most discriminate single regions, which are the left and right posterior callosomarginal fissures.

The aim of this study was to implement a machine learning tool that automatically identifies NPH-typical MRI features (NPH pattern) on routinely acquired MRI sequences.

## Methods

Patients: presurgical MRI scans of 30 NPH patients, who had been treated with a ventriculoperitoneal shunt were retrospectively compared to an age and gender-matched group of 30 healthy controls (HC). Surgery had been performed between 2010 and 2017, and patients re-evaluated several times after shunt implantation thereafter. We screened our neurosurgical database retrospectively for patients first time receiving a CSF shunt due to a NPH between 2010 and 2017. As a second step, we included those having undergone 3D T1-weighted MRI scans.

Readings: two senior (26 and 19 years of experience) and two junior neuroradiologists (4 and 2 years of experience) independently assessed the presence of a NPH pattern on a 3D sagittal magnetization prepared rapid acquisition gradient recalled echo (MP-RAGE) sequence with isotropic voxels (1 × 1 × 1 mm^3^). They were asked to assess imaging features, such as the DESH sign, THC and small callosal angle and to finally grade them on a 3-point scale (0 = no NPH pattern, 1 = possible NPH pattern, 2 = definite NPH pattern). Anonymized imaging data of patients and controls were shown in a randomized order. In addition, MRI reports were retrospectively reviewed whether NPH features were described or the condition was dismissed, e.g. as unspecific ventriculomegaly.

**Fig. 1:**
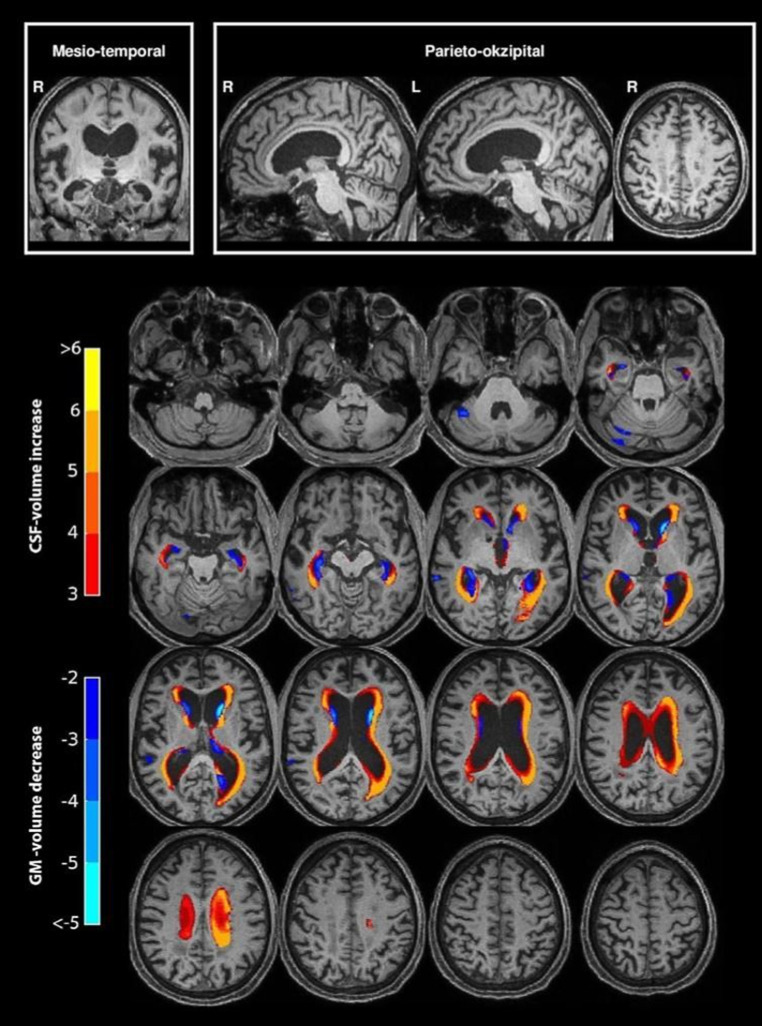
Support vector machine (SVM) results showing abnormal gray matter (GM) and cerebrospinal fluid (CSF) regions superimposed in color on a normal pressure hydrocephalus (NPH) patient’s (82, male) individual 3D T1-weighted brain MRI in serial sections from caudal to cranial (increase of CSF volume in *red to yellow*, and decrease of GM volume in *dark to light blue*). On the left color bars indicate the corresponding z‑scores. SVM analysis came to a prediction score of 0.94. Additionally, representative brain slices (from *left* to *right*: coronary, parasagittal right and left, axial close to the convexity) are presented in the top row with a typical disproportionately enlarged subarachnoidal space hydrocephalus (DESH) sign in this specific NPH case (*top left*)

Machine learning: for NPH identification, a linear support vector machine (SVM) was employed [13]. A SVM is a supervised machine learning tool in which a single case is grouped to one of two categories (here: NPH pattern—no NPH pattern). Groups of 30 NPH patients and 30 HC each were taken to train the algorithm, making it a non-probabilistic binary linear classifier. The NPH patients and HC are thus mapped as points in a multidimensional space, so that both groups are separated by a clear gap that is as wide as possible. New cases are then mapped into that same space and predicted to belong to a category of “NPH pattern” or “no NPH pattern”. We used T1-weighted MP-RAGE scans with features being constructed based on SPM (Statistical Parametric Mapping) 12 segmentation and normalization procedures [14, 15]. Gray matter (c1-map) and CSF (c3-map) segmentations were warped into the MNI (Montreal Neurological Institute) space (using modulation of gray value by the Jacobian of the warp) and smoothed by full width half max 3 × 3 × 3 mm, similar to what is done for usual voxel-based morphometry analyses [16, 17]. The volume of gray matter (GM) and CSF of 272 regions defined in a probabilistic brain atlas (http://www.neuromorphometrics.com/) were extracted as a direct input into the SVM (MATLAB 2018a, standard parameter setting). Fivefold cross-validation was performed. The SVM results are given as a prediction score from 0 to 1. An example for an analysis is displayed in Fig. 1.

Statistical analysis: SVM performance was compared to human ratings in terms of correctly predicting NPH cases. Standard metrics for binary classification such as accuracy, sensitivity, specificity and negative prediction value were calculated. A receiver operating characteristic (ROC) analysis displayed sensitivity and specificity of SVM in identifying NPH patients. Interobserver agreement was assessed by Fleiss’ κ [18].

## Results

A total of 30 patients were included in the NPH group and 30 HC served as control group. Clinical characteristics are displayed in Table 1.

**Table 1:** Patient characteristics

	Patients (*n* = 30)		Healthy controls (*n* = 30)
Age (years)	75.8 (range 67–86)		72.9 (range 65–84)*
Sex (male)	20 (66.7%)		20 (66.7%)**
Clinical presentation	Gait disturbance	30 (100%)	–
	Cognitive decline	22 (73.3%)	
	Urine incontinence	18 (60%)	
	Full Hakim triad	14 (46.7%)	
	Gait disturbance only	2 (6.7%)	
Follow-up	No benefit after shunting	6 (of *n* = 28) (21.4%)	–

Population characteristics for the dataset used in this study. Summaries are given as count (percent)

Groups were not different in terms of age *(*p* = 0.11) and sex **(*p* = 1)

Patients were clinically re-evaluated directly after and 3 months after surgery (mean 57 days). One patient was lost in follow-up and another patient died after surgery (after initial NPH symptom reduction) due to acute respiratory distress syndrome. After shunting, 20 patients (of 28 patients evaluated in follow-up) showed a marked improvement of NPH symptoms. In two patients, improvement was unclear, six patients did not benefit.

**Fig. 2:**
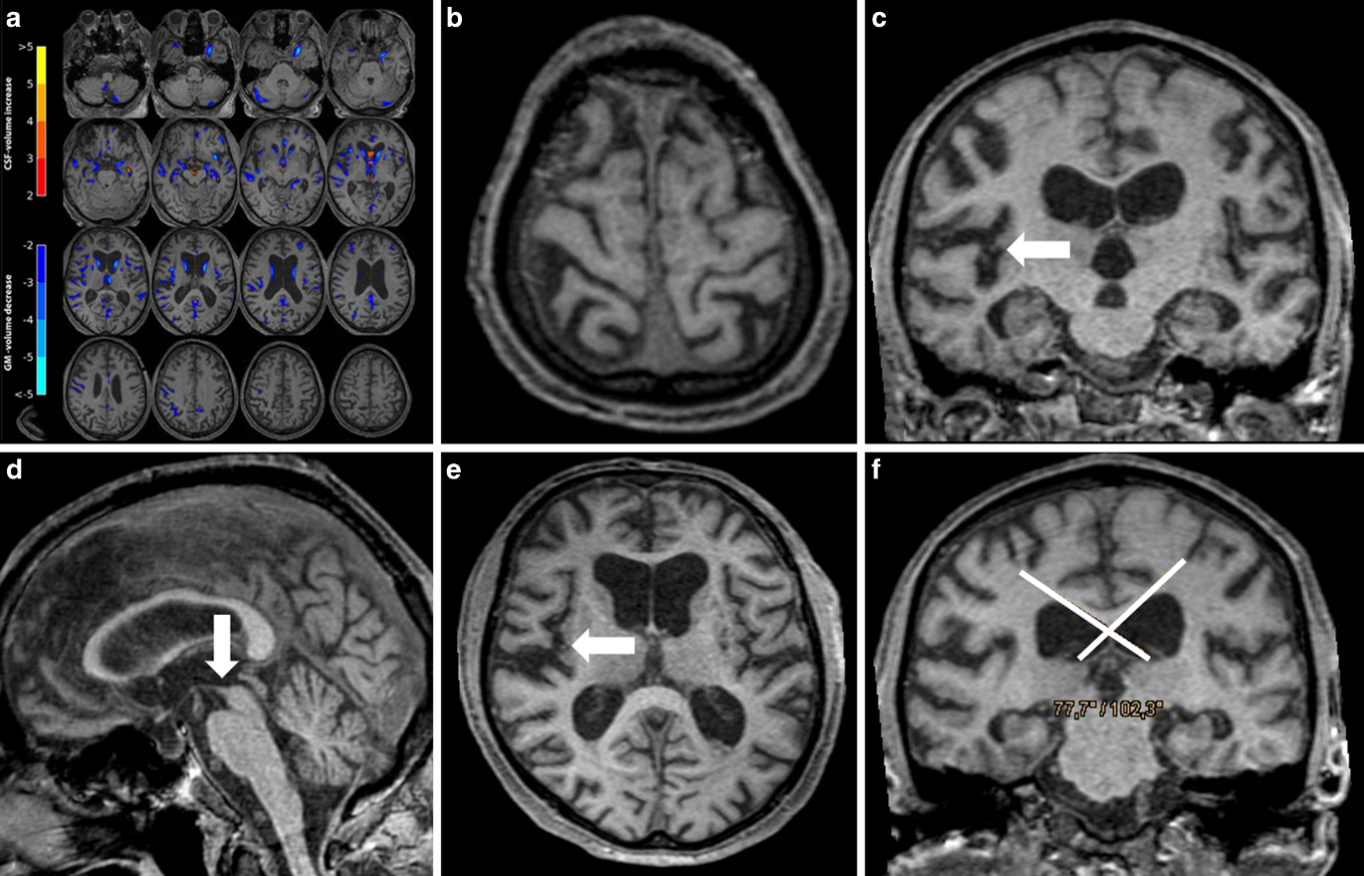
**a** SVM analysis as described in Fig. 1. Volume changes are pronounced in the right Sylvian fissure, but definite CSF volume increase in the ventricles is missing. Nevertheless, SVM analysis assigned a prediction score of 0.96. **b–f** Representative images of the same patient (74 male) displaying a tight high convexity (**b**), widening of the right Sylvian fissure (*arrows* in **c,** **e**), dilatation of the third ventricle mimicking a Colibri sign (*arrow* in **d**) and a callosal angle of 103° (*angle* in **f**). Note that the callosal angle in NPH is typically < 90°. Both junior raters assessed “no NPH pattern”, both senior raters a “probable NPH pattern”

Human readings are displayed in Table 2. Junior raters assigned a “no NPH” pattern in three and six NPH patients, and senior raters in one and two patients, respectively. An example of a “probable NPH” pattern not identified by the junior raters is given in Fig. 2. Interobserver reliability was substantial with a κ-value of 0.656 (*p* < 0.001).

**Table 2:** Individual ratings of the human readers

	Rater 1 (Senior)		Rater 2 (Senior)		Rater 3 (Junior)		Rater 4 (Junior)	
“No NPH pattern”	2 (6.7%)		1 (3.3%)		6 (20%)		3 (10%)	
“Possible NPH”	6 (20%)		6 (20%)		5 (16.7%)		7 (23.3%)	
“Definite NPH”	22 (73.3%)		23 (77.7%)		19 (63.3%)		20 (66.7%)	
Healthy controls	No NPH pattern	27 (90%)	No NPH pattern	27 (90%)	No NPH pattern	30 (100%)	No NPH pattern	29 (97%)
	Possible NPH	3 (10%)	Possible NPH	3 (10%)	–	–	Possible NPH	1 (3%)

Total amount (percentage)

*NPH* normal pressure hydrocephalus

**Fig. 3:**
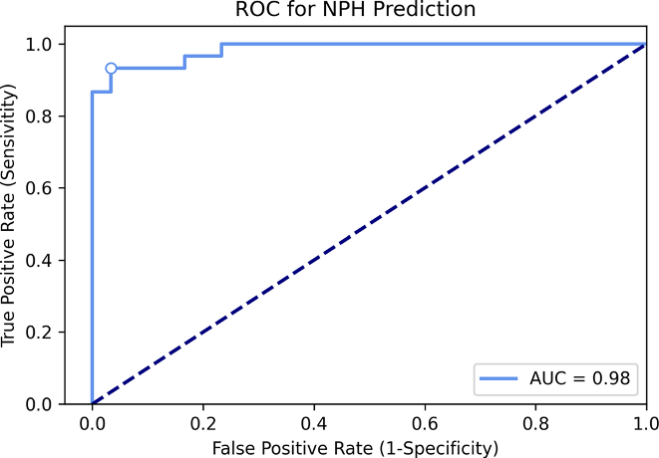
The receiver operating characteristic (*ROC*) curve displays the performance of the support vector machine (SVM) in terms of identifying normal pressure hydrocephalus (*NPH*) patients

SVM performance: with a prediction score of 0.42, acceptable performance was achieved with one false negative and three false positive results. Human readings and SVM results are compared in Table 3. In the one false negative NPH case (prediction score of 0.37), a “no NPH pattern” was observed by all human readers. This patient had presented with gait disturbance and dementia and improved upon shunt implantation. The ROC (Fig. 3) shows the SVM reliably identified all NPH patients with an AUROC of 0.98.

**Table 3:** Statistics

Possible and definite “NPH pattern”	Rater 1 (Senior)	Rater 2 (Senior)	Rater 3 (Junior)	Rater 4 (Junior)	SVM
Accuracy	0.92	0.93	0.9	0.93	0.93
Sensitivity	0.93	0.96	0.83	0.91	0.90
Specificity	0.90	0.91	1.0	0.96	0.96
NPV	0.93	0.97	0.8	0.9	0.90

Performance of human reading and SVM in detection of NPH patients

*NPH* normal pressure hydrocephalus, *SVM* support vector machine, *NPV* negative predictive value

**Fig. 4:**
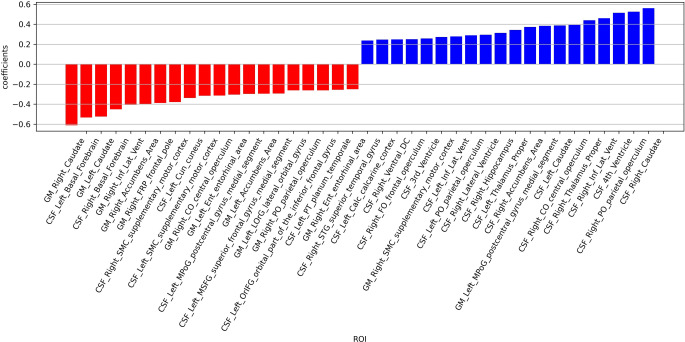
Depiction of the regions with the highest power to separate a NPH (normal pressure hydrocephalus) and a no NPH pattern among the 272 prespecified regions. The discriminative power of the regions is plotted with respect to GM (grey matter; *red*) and CSF (cerebrospinal fluid, *blue*)

With respect to the discriminative power of the 272 regions defined in the probabilistic brain atlas, the following 5 regions served best to separate NPH and healthy controls: right caudate, right parietal operculum, left basal forebrain, left caudate and 4th ventricle (Fig. 4).

## Discussion

A NPH pattern can be reliably detected by a SVM algorithm processing 3D T1-weighted datasets which are routinely acquired in many of our patients with neurological complaints.

The NPH patients in our cohort had typical clinical symptoms and ages, and a reasonable number were shunt responders. Although more males than females were treated, we consider our cohort as a typical NPH cohort [5, 19, 20].

In almost all NPH patients, even junior neuroradiologists correctly identified a definite NPH pattern. Of note is that the readers in this study were trained to identify a NPH pattern and knew patients had received a CSF shunt due to NPH. Nevertheless, in only 19 of the 30 NPH patients the NPH pattern had been described in the initial MRI reports. Here, an SVM-aided diagnosis may significantly change clinical management. Considering the accuracy of 0.93 and the AUROC of 0.98, SVM is suited for clinical practice. Given the high prevalence of NPH in old patients, one may presume an increase of NPH pattern reports. As the radiological features may precede clinical symptoms the automatic detection during the asymptomatic stage could help to prevent dementia and associated costs in these patients [3, 21]. Other groups have already focused on automated NPH detection. Unspecific ventricular enlargement was successfully detected in computed tomography (CT) imaging [22] and machine learning was used to determine most discriminate regions in patients who had been identified by radiologists as having a NPH pattern [12]. In contrast, we compared the performance of an SVM against human readings in terms of how reliably NPH patients can be identified in MRI and surpassed the accuracy of others who differentiated NPH, vascular cognitive disease and HC using sulcal patterns [23] or a 3D convolutional ladder network in the differentiation of NPH, Alzheimer’s dementia and HC [24]. Zhang et al. described a similar approach on CT imaging with a comparable group (27 NPH patients and 34 HC) and reached acceptable sensitivity, too [25]. A strength of this approach is that it does not focus on a single region but uses the overall pattern of GM and CSF spaces. The SVM showed both caudate, the right parietal operculum, the left basal forebrain, and the 4th ventricle as most discriminative of 272 regions to separate a NPH and a no NPH pattern. That caudate and the parietal operculum have altered CSF volumes sounds reasonable. The CSF volume changes of the (left) basal forebrain, however, likely also occur to the atypical configuration of the CSF spaces.

Concerning false positives, two HC were considered to have a definite NPH pattern by both senior raters and the SVM. This number matches the estimated prevalence of a NPH pattern in this age group [3, 21].

Another goal of imaging is to identify possible shunt responders. The SVM prediction score could not differentiate between responders and non-responders. Prediction of shunt responsiveness is difficult as it depends on multiple factors, such as evolution of clinical symptoms, duration since symptom onset, and likely requires a larger study group as retrospective data were not detailed enough [6]; however, it has already been shown that individual NPH features, such as the callosal angle or THC correlate with the outcome [26, 27] so that futile shunting and its complications could be prevented.

Despite the low number of patients and the retrospective data collection this single-center proof of concept study shows that NPH patients can be automatically detected with a high accuracy similar to human ratings. This detection is helpful as a NPH pattern often interferes with voxel-based morphometry to detect region-specific atrophy [28]; however, use needs to be confirmed by a larger and more structured data assessment. In this context, the comparison of the SVM prediction score with a recently published visual imaging score (iNPH Radscale) would be worthwhile [29]. In the iNPH Radscale, eight features such as the Evans index, narrow sulci, Sylvian fissures, focally enlarged sulci, widths of temporal horns, callosal angle, and periventricular hypodensities are visually assessed and converted into points from 0 to 12. Excluding white matter hypodensities the former features are likely to be “considered” by the SVM approach as it processes GM and CSF probability maps in 272 regions.

## Conclusion

Considering the high prevalence of normal pressure hydrocephalus (NPH) in old patients and rising numbers of MRI scans in this group, one can presume an increased incidence of a NPH pattern. Since the NPH pattern may precede clinical symptoms, automatic detection during a presymptomatic stage could be a promising tool for an early diagnosis. Trained machines such as the presented support vector machine (SVM) might simplify diagnosis with no apparent loss of reliability compared to human performance.

## Abbreviations

CSF: Cerebrospinal fluid
DESH: Disproportionately enlarged subarachnoid space hydrocephalus
GM: Gray matter
HC: Healthy controls
NPH: Normal pressure hydrocephalus
SVM: Support vector machine
THC: Tight high convexity
